# Hypothetical interventions on emergency ambulance and prehospital acetylsalicylic acid administration in myocardial infarction patients presenting without chest pain

**DOI:** 10.1186/s12872-022-03000-1

**Published:** 2022-12-22

**Authors:** Amalie Lykkemark Møller, Helene Charlotte Wiese Rytgaard, Elisabeth Helen Anna Mills, Helle Collatz Christensen, Stig Nikolaj Fasmer Blomberg, Fredrik Folke, Kristian Hay Kragholm, Freddy Lippert, Gunnar Gislason, Lars Køber, Thomas Alexander Gerds, Christian Torp-Pedersen

**Affiliations:** 1 Department of Cardiology, Nordsjællands Hospital, Hillerød, Denmark; 2 Section of Biostatistics, Department of Public Health, University of Copenhagen, Copenhagen, Denmark; 3 Department of Cardiology, Aalborg University Hospital, Aalborg, Denmark; 4 Danish Clinical Quality Program (RKKP), National Clinical Registries, Frederiksberg Hospital, Frederiksberg, Denmark; 5 Copenhagen Emergency Medical Services, Ballerup, Denmark; 6 Department of Cardiology, Copenhagen University Hospital Herlev and Gentofte, Hellerup, Denmark; 7 Department of Clinical Medicine, University of Copenhagen, Copenhagen, Denmark; 8 Unit of Clinical Biostatistics and Epidemiology, Department of Cardiology, Aalborg University Hospital, Aalborg, Denmark; 9 Department of Research, Danish Heart Foundation, Copenhagen, Denmark; 10 The National Institute of Public Health, University of Southern Denmark, Copenhagen, Denmark; 11 Department of Cardiology, Rigshospitalet Copenhagen University Hospital, Copenhagen, Denmark

**Keywords:** Myocardial infarction, Acetylsalicylic acid, Emergency medical services, Prehospital management, Symptom

## Abstract

**Background:**

Myocardial infarction (MI) patients presenting without chest pain are a diagnostic challenge. They receive suboptimal prehospital management and have high mortality. To elucidate potential benefits of improved management, we analysed expected outcome among non-chest pain MI patients if hypothetically they (1) received emergency ambulances/acetylsalicylic acid (ASA) as often as observed for chest pain patients, and (2) all received emergency ambulance/ASA.

**Methods:**

We sampled calls to emergency and non-emergency medical services for patients hospitalized with MI within 24 h and categorized calls as chest pain/non-chest pain. Outcomes were 30-day mortality and a 1-year combined outcome of re-infarction, heart failure admission, and mortality. Targeted minimum loss-based estimation was used for all statistical analyses.

**Results:**

Among 5418 calls regarding MI patients, 24% (1309) were recorded with non-chest pain. In total, 90% (3689/4109) of chest pain and 40% (525/1309) of non-chest pain patients received an emergency ambulance, and 73% (2668/3632) and 37% (192/518) of chest pain and non-chest pain patients received prehospital ASA. Providing ambulances to all non-chest pain patients was not associated with improved survival. Prehospital administration of ASA to all emergency ambulance transports of non-chest pain MI patients was expected to reduce 30-day mortality by 5.3% (CI 95%: [1.7%;9%]) from 12.8% to 7.4%. No significant reduction was found for the 1-year combined outcome (2.6% CI 95% [− 2.9%;8.1%]). In comparison, the observed 30-day mortality was 3% among ambulance-transported chest pain MI patients.

**Conclusions:**

Our study found large differences in the prehospital management of MI patients with and without chest pain. Improved prehospital ASA administration to non-chest pain MI patients could possibly reduce 30-day mortality, but long-term effects appear limited. Non-chest pain MI patients are difficult to identify prehospital and possible unintended effects of ASA might outweigh the potential benefits of improving the prehospital management. Future research should investigate ways to improve the prehospital recognition of MI in the absence of chest pain.

**Supplementary Information:**

The online version contains supplementary material available at 10.1186/s12872-022-03000-1.

## Background

Despite advances in the treatment and survival of patients with myocardial infarction (MI) [1, 2], both short and long-term mortality remains high among patients presenting without the cardinal symptom of chest pain [3, 4]. MI patients presenting without chest pain have been found to have longer prehospital delays [5, 6], to be older, have more comorbidities, and use more medication than MI patients with chest pain [3]. A focus on how to improve treatment of survival of this high-risk patient group is necessary.

Effective treatment of MI requires timely intervention and potential life-saving efforts that can be implemented prior to hospitalization including the immediate dispatch of an ambulance to patients and provision of acetylsalicylic acid (ASA) upon arrival of the ambulance [7, 8]. No randomized trials have assessed the effect of increasing ambulance use and prehospital ASA administration among non-chest pain MI patients. An observational study of MI patients found that patients administered aspirin prehospital had a lower observed 1-year mortality than those who did not receive it [9]. Similarly, early administration of ASA versus late have been found to improve 7-day and 30-day survival among STEMI patients in other observational studies [10, 11]. However, the certainty of the evidence from these studies was evaluated to be very low [12]. Emergency dispatch priority, history of myocardial infarction, and chest pain perceived as of cardiac origin have been found to be associated with ASA administration [9, 13]. Further, chest pain was highly associated with prehospital suspicion of ischemic heart disease which again was closely linked to ASA administration [9]. As previous studies indicate that just 45–58% of acute coronary syndrome/MI patients receives prehospital ASA, improving prehospital use of ASA could be an important target to reduce mortality, especially among the MI patients who present without the cardinal symptom of chest pain [9, 14, 15]. Therefore, to examine whether the additional focus should be on the emergent medical services and treatment with ASA we have used a causal inference framework to study whether early intervention by ambulance dispatch and ASA treatment might potentially benefit MI patients presenting without chest pain. We have used this framework to test whether emergency ambulance dispatch and/or ASA therapy administered to all or to the same proportion as observed for MI patients with chest pain is likely to benefit MI patients without chest pain. Since this study focuses on patients that eventually are diagnosed with an MI we cannot address any adverse effects of providing ASA or emergency service to patients without an infarction. The study should be regarded as a test to whether potential positive effects of improved prehospital management exist. Results from this study can be used to assess if further studies of prehospital intervention in patients with atypical symptoms of myocardial infarction are warranted, including assessments of the economic and administrative burden of increased ambulance dispatch and adverse effects of ASA administration.

In consequence, we have examined a large dataset of calls to an emergency and a non-emergency medical service in Copenhagen, Denmark, where prehospital care and emergency ambulance dispatches are recorded. We investigated whether MI patients without chest pain could be expected to benefit from increased emergency ambulance dispatch and prehospital ASA treatment.

## Methods

### Setting

In Copenhagen, Denmark, citizens having a medical emergency should dial 1-1-2, equivalent to 9-1-1 in the United States, to receive assistance. At the 1-1-2 emergency number, the call takers are paramedics and nurses, and they assess the medical urgency and prioritize dispatching of the ambulances. Citizens with non-emergency medical conditions can contact their general practitioner or call 1813 to reach the regional 24-h medical helpline (the 1813-medical helpline) which is intended to cover outside the working hours for general practitioners. Here, primarily nurses, but also medical doctors provide medical guidance, refer to emergency departments, and dispatch ambulances. The 1813-medical helpline and the 1-1-2 emergency number is a combined medical service operated by Copenhagen Emergency Medical Services, serving 1.8 million inhabitants, approximately one-third of the Danish population [16]. Although the 1813-medical helpline and the 1-1-2 emergency number are two separate services, they are physically located in the same building and both services use the same integrated software system. In Denmark, the health care system is tax-funded, and no user fees apply to emergency and non-emergency medical services as well as hospital treatments.

### Data and variables

In Denmark, all citizens with permanent residency are assigned a unique civil registration number. This number is registered when calling for help at the 1813-medical helpline and 1-1-2 emergency number and in most other encounters with the public sector [17, 18].

We retrieved information on age, sex, ethnicity (Danish and immigrant/descendant of immigrant), and country of emigration from the Danish Civil Registration System. From the Population Educational Registers [19] patients’ highest achieved education were classified into three groups based on the International Standard Classification of Education values (ISCED) (0-2: Basic, 3: Intermediate, and 5-8: Advanced). ISCED level 4 is not used in Denmark. Comorbidities, including ischemic heart disease, MI, Chronic Obstructive Pulmonary Disease (COPD), and heart failure were defined by the International classification of disease codes (ICD-10) registrations of a primary or secondary diagnosis in the Danish National Patient Registry [20] up to 5 years prior to the call. The Danish National Prescription Registry [21] was used to define type 2 diabetes, hypertension, Non-Steroid Anti-Inflammatory Drugs (NSAID), and opioid use, all within 180 days of the call. Type 2 diabetes was defined as a claimed prescription of hypoglycaemic medication and hypertension was diagnosed if at least two classes of antihypertensive drugs had been claimed, both at most 180 days before the call [22]. A full list of ICD-10 codes and Anatomical Therapeutic Chemical Classification System (ATC) codes used to define comorbidity and medicine use is available in Additional file 1: Table S1 and S2. Information on the medical service the patient contacted, the criterion (symptom/description), and vehicle assignment was drawn from the computer-assisted dispatch system (CAD—Logis Solutions) at The Copenhagen Emergency Medical Services. Prehospital assigned treatment and contraindication of treatment were defined using the electronic prehospital patient record, in which ambulance personnel record observations and treatment related to the patient during transportation [23].

### Population

All contacts for patients above age 30 to the prehospital services in Copenhagen (1813-medical helpline and 1-1-2 emergency number) between April 1st, 2015, and December 31st, 2018 were considered. We included calls regarding patients hospitalized with a confirmed MI no later than 24 h after the call. We defined MI as a primary diagnosis of ICD-10 I21 identified from the Danish National Patient Registry. The same patients can occur multiple times in the study population, given that the same patient could; have experienced more than one MI during the study period, or have more than one call to the Copenhagen Emergency Medical Service within the 24-h time span. We excluded patients who were terminal, living at nursing homes, suspected dead at the time of call, unconscious, and calls with missing criteria or criteria that were non-informative of the patient's condition. For the analysis of prehospital ASA assignment, only MI patients who received an emergency ambulance response were included. Additionally, we excluded patients with contraindications for ASA treatment, defined by occurrence of any of the following words in the electronic prehospital medical record: ‘blood’, ‘bleeding’, ‘aneurysm’, or ‘ulcer’, or notation of allergy to ASA.

### Registration of symptoms

Health care professionals at the 1813-medical helpline and 1-1-2 emergency number choose one criterion (symptom/description) to describe the situation/symptoms of the patients using criteria-based systems. The criteria are used for registration but also for the assigning of vehicles. Notably, the two services use different protocols. A locally developed electronic decision support system is used at the 1813-medical helpline and Danish Index is used at the 1-1-2 emergency number [24]. For this study, the assigned criteria are proxy of the patient’s symptoms and will be referred to as such. Prior to the analysis symptoms were categorized into chest pain and other symptoms (non-chest pain). Non-chest pain included breathing problems, abdominal/back/urinary symptoms, infection/fever, trauma, unclear problem, and impaired consciousness/dizziness among others.

### Emergency ambulance and prehospital acetylsalicylic acid

This study aims to investigate if the excess mortality observed among MI patients with non-chest pain could be reduced if we hypothetically intervened on two prehospital factors, (1) receiving an emergency ambulance and (2) receiving prehospital ASA treatment. Receiving an emergency ambulance was defined as the dispatch of an emergency ambulance with lights and sirens, which is a mobile intensive care unit of type A. The service aim of emergency ambulances in Copenhagen, Denmark is 90% arriving at the patient within less than 13 min. All other dispatches including non-emergency ambulances, self-transport to hospital, home visits were defined as no emergency ambulance. Further details of the remaining types of dispatches/non-dispatches are available [25, 26].

Our secondary aim was to investigate the prehospital management of MI patients after they received emergency ambulances. Prior to data access, ASA was chosen as the intervention of interest, as ASA is recommended as the prehospital treatment of MI patients regardless of later in-hospital treatment [7, 27]. Prehospital ASA treatment was defined as the registration of ASA (predominantly oral administration but also intravenous) in the medicine tab of the electronic prehospital medical record.

### Outcomes

The primary outcome was 30-day all-cause mortality, and the secondary outcome was a 1-year combined outcome. We defined 30-day mortality from The Danish Civil Registration System [17, 18] and included all deaths occurring between the time of MI diagnosis and 30 days after. The 1-year combined outcome was defined as any occurrence of death or hospitalization with either re-infarct or heart failure within 1 year of the MI diagnosis, but not including MI or heart failure diagnosed during the same hospital admission as the MI for which the patient was included in this study.

### Statistics

All analyses were repeated for both outcomes. We defined effect measures in a causal inference framework [28] in terms of expected outcome risk under hypothetical interventions on dispatch of emergency ambulances and prehospital ASA treatment.

Four main analyses were performed as outlined in Table 1.

**Table 1:** Overview of the hypothetical interventions and study populations considered in the analyses

Main analyses*	Abbreviation	Study population	Hypothetical intervention
First	As chest pain	All non-chest pain MI patients	Non-chest pain MI patients received *emergency ambulances* as observed for MI patients with chest pain
Second	All		All non-chest pain MI patients received *emergency ambulances*
Third	As chest pain	All non-chest pain MI patients who received an emergency ambulance and did not have contraindications of ASA	Non-chest pain MI patients received *prehospital ASA* as observed for MI patients with chest pain
Fourth	All		All non-chest pain MI patients received *prehospital ASA*

*All analyses were repeated in subgroups of STEMI and NSTEMI patients, and calls to the 1-1-2 emergency number. The target of the hypothetical intervention is shown in *italic*. *ASA* Acetylsalicylic acid

In the first and third analysis, we estimated the interventional disparity indirect effect [29] among non-chest pain MI patients, a variant of interventional effects proposed by VanderWeele, Vansteelandt and Robins [30]. A small distinction is that we standardized the outcome risk to the covariate distribution among the target population, i.e. non-chest pain MI patients, to have a more clinically relevant interpretation. In the first analysis, we estimated the expected outcome risk among non-chest pain MI patients if, hypothetically, they had received an emergency ambulance with the same probability as observed for MI patients with chest pain (*as chest pain*-intervention). In the second analysis, we estimated the expected outcome risk if all MI patients recorded without chest pain had received an emergency ambulance, referred to as the *all-*intervention. Both hypothetical interventions were compared to the observed outcome risk in non-chest pain MI patients and our target parameter for each analysis was the expected reduction in outcome risk under the hypothetical intervention of interest. The third and fourth analyses considered only patients who received an emergency ambulance and did not have contraindications towards ASA. In this subgroup, the third analysis targeted the expected outcome risk had non-chest pain MI patients received ASA treatment with the same probability as observed for chest pain patients (*as chest pain*-intervention), and the fourth analysis targeted the expected outcome probability had all non-chest pain MI patients received ASA treatment (*all*-intervention). Similarly, these hypothetical interventions were compared to the observed outcome risk among non-chest pain MI patients who received an emergency ambulance and our target parameter was the expected reduction in outcome risk under the hypothetical interventions of interest.

The target parameters in all analyses were estimated using targeted minimum loss-based estimation (TMLE) [31]. Initial estimates for the TMLE were obtained based on logistic regression for the binary outcomes, the probability of having chest pain, and the probability of emergency ambulance dispatch or ASA treatment. The logistic regression models were fitted using discrete super learner [32], where super learning (a cross-validation based method) is used to select the best performing algorithm among candidate algorithms. The candidate algorithms included logistic regression models with and without second-order interaction terms and were adjusted for age, sex, medical service (1813-medical helpline or 1-1-2 emergency number), type-2 diabetes, heart failure, chronic obstructive pulmonary disease (COPD), opioid use, previous myocardial infarction, and educational level. In addition, some regression models were adjusted for cancer, immigration, and ischemic heart disease. Regression models of the outcome (30-day mortality and 1-year combined outcome) were also adjusted for type of infarction.

All analyses were repeated in three subgroups: STEMI patients, NSTEMI patients, and among calls to the 1-1-2 emergency number. STEMI and NSTEMI were defined according to ICD-10 codes registered during the hospitalization (See Additional file 1: Table S3). Further, we estimated the observed mortality and 1-year combined outcome in MI patients with chest pain and assessed outcome risks had they all received emergency ambulances and ASA, respectively. In these analyses we standardized to the covariate distribution among chest pain MI patients. The level of statistical significance was set at 5%. The TMLE used for this study is implemented in an R package available at GitHub (https://github.com/amalielykkemark/tmleExposed). All analyses were performed in R version 4.0.3 [33].

## Results

**Fig. 1:**
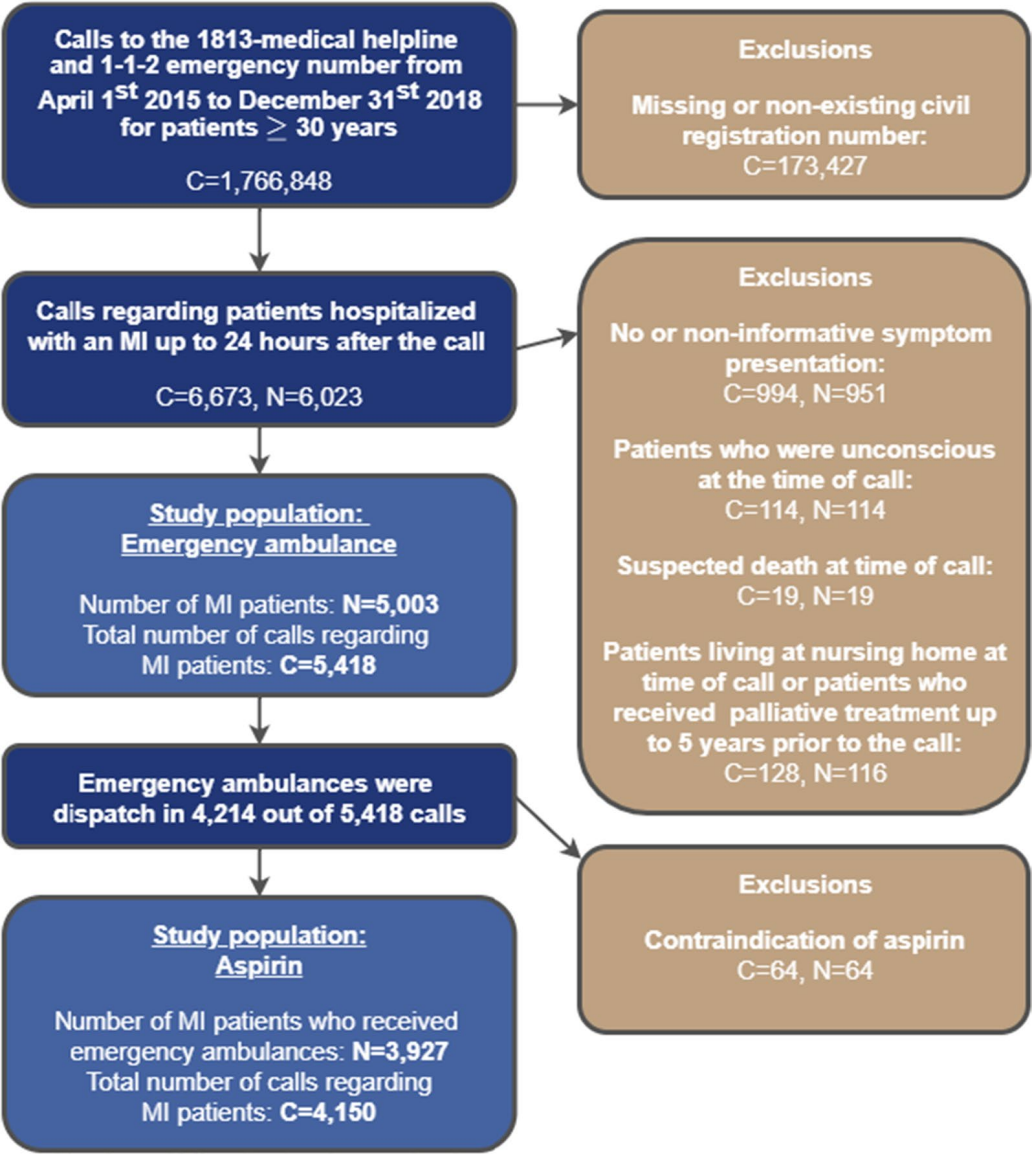
Flowchart of the study population, *C* Number of calls, *N* Number of unique MI patients

In total, 1,766,848 calls were registered at the 1813-medical helpline and 1-1-2 emergency number from April 1st, 2015 to December 31st, 2018 for patients aged 30 or above, of which 1,593,421 had information of civil registration number. We identified 6673 calls regarding 6023 patients who received a hospital diagnosis of MI within 24 h of the call. We excluded calls regarding patients; living at nursing homes, in palliative care, suspected dead at the time of call, who were unconscious, and calls with missing information or non-informative symptom presentation, resulting in 5418 calls regarding 5003 MI patients. Further details of the exclusions are available in Fig. 1.

### Patient characteristics

Characteristics of the MI population are shown according to the recorded symptom presentation in Table 2. Among the 5418 MI patients, 4109 (75.8%) were recorded with chest pain, and 1309 (24.2%) were recorded without chest pain as the primary symptom. In total 65.7% were male, the median age was 69 IQR[57.8, 77.9] and 34.9% had called the 1813-medical helpline in relation to their MI. The MI patients recorded without chest pain were older (median 73.5 years vs 67.5 years), more likely to have basic education as highest attained education (38.0% vs. 31.1%), had a bigger burden of comorbidities, with a high prevalence of COPD (14.6% vs. 6.1%), more likely to have redeemed an opioid prescription (24.8% vs. 15.0%) and were more likely to have called the 1813-medical helpline (45.5% vs. 31.5%) compared to MI patients with chest pain. In contrast, MI patients with chest pain had a higher prevalence of previous ischemic heart disease (27.3%) and myocardial infarction (18.2%) compared to non-chest pain MI patients (IHD: 19.5% MI: 11.5%) (Table 2). During the hospital admission with MI, for which the patients were included in this study, non-chest pain MI patients were more often registered with heart failure (21.5% vs. 10.3%), COPD (9.6% vs. 2.2%), and cardiogenic shock (2.1% vs. 0.6%) compared to chest pain MI patients (See Additional file 1: Table S4).

**Table 2:** Patient characteristics of MI patients recorded with and without chest pain

Variable	Level	Chest pain (n = 4109)	Non-chest pain (n = 1309)	Total (n = 5418)
Medical services	1813-medical helpline n(%)	1296 (31.5)	596 (45.5)	1892 (34.9)
	1-1-2 emergency number n(%)	2813 (68.5)	713 (54.5)	3526 (65.1)
Sex	Female n(%)	1280 (31.2)	579 (44.2)	1859 (34.3)
	Male n(%)	2829 (68.8)	730 (55.8)	3559 (65.7)
Age	median [iqr]	67.5 [56.6, 76.5]	73.5 [62.8, 81.9]	69 [57.8, 77.9]
Educational level	Basic n(%)	1215 (31.1)	468 (38.0)	1683 (32.8)
	Intermediate n(%)	1747 (44.8)	512 (41.6)	2259 (44.0)
	Advanced n(%)	941 (24.1)	251 (20.4)	1192 (23.2)
	Missing n*	206	78	284
Ethnicity	Danish n(%)	3461 (84.2)	1096 (83.7)	4557 (84.1)
	Immigrant/descendant of immigrant n(%)	642 (15.6)	210–213 (16)	852–855 (15–16)
	Missing n **	6	0–3	6–9
Type 2 diabetes	n(%)	644 (15.7)	230 (17.6)	874 (16.1)
Hypertension	n(%)	1385 (33.7)	482 (36.8)	1867 (34.5)
Opioids	n(%)	616 (15.0)	324 (24.8)	940 (17.3)
NSAID	n(%)	524 (12.8)	187 (14.3)	711 (13.1)
Previous ischemic heart disease	n(%)	1123 (27.3)	255 (19.5)	1378 (25.4)
Previous myocardial infarction	n(%)	749 (18.2)	150 (11.5)	899 (16.6)
Previous heart failure	n(%)	422 (10.3)	149 (11.4)	571 (10.5)
Previous cancer (excluding malign melanoma)	n(%)	367 (8.9)	142 (10.8)	509 (9.4)
Previous moderate/severe renal disease	n(%)	207 (5.0)	88 (6.7)	295 (5.4)
Previous COPD	n(%)	249 (6.1)	191 (14.6)	440 (8.1)
Previous atrial fibrillation	n(%)	401 (9.8)	147 (11.2)	548 (10.1)
Allergy to ASA/contraindication (excluded from the analysis of ASA)	n(%)	59 (1.4)	7 (0.5)	66 (1.2)

*Missing educational level was recoded to Basic education for analyses. **Missing ethnicity was recoded to Immigrant/descendant of immigrant for analyses. *NSAID* Nonsteroidal anti-inflammatory drugs, *COPD* Chronic obstructive pulmonary disease. *ASA* Acetylsalicylic acid

**Fig. 2:**
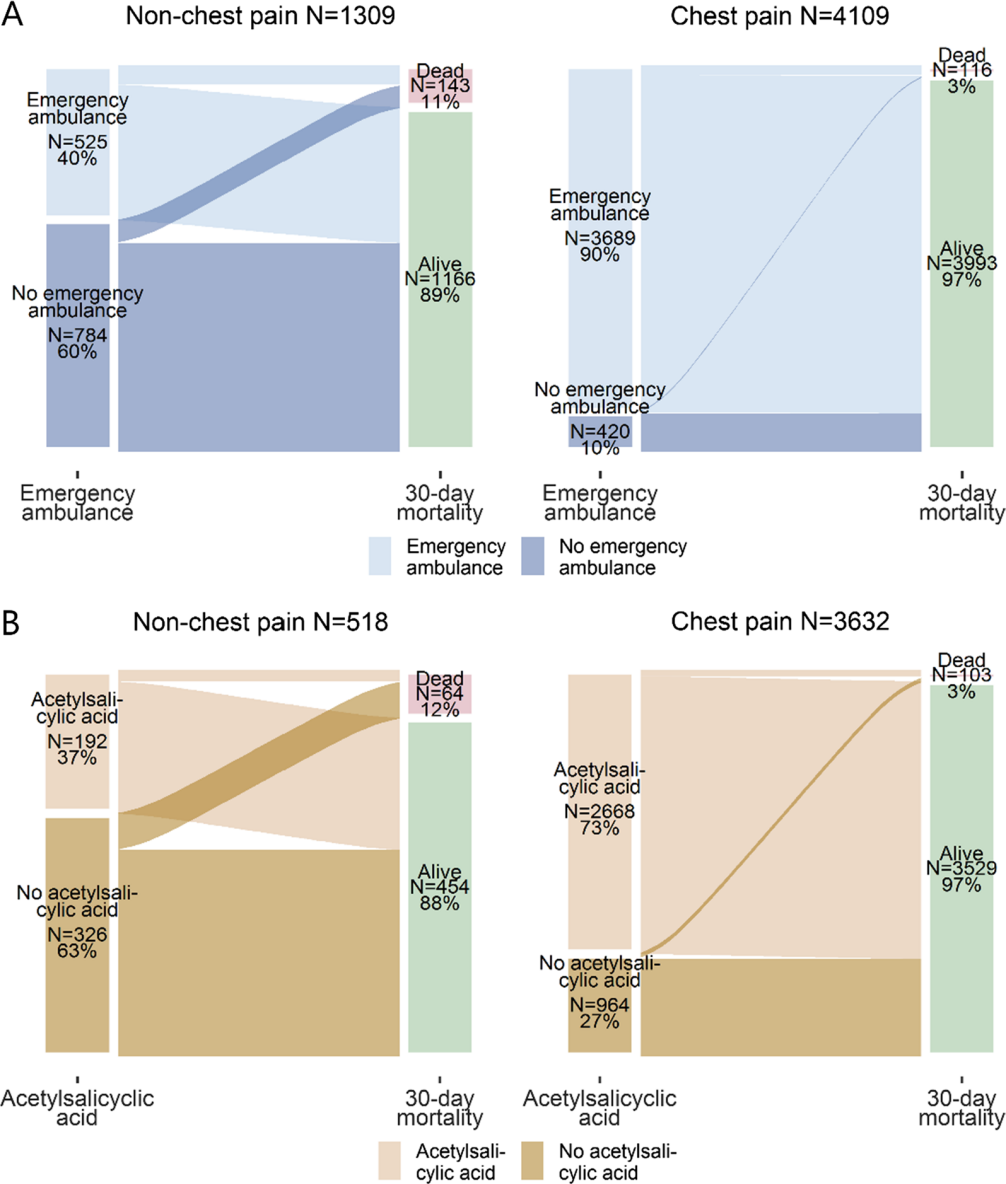
Emergency ambulance dispatch, prehospital ASA administration, and mortality by recorded symptom. **A** A total of 5418 MI patients were included. The median time from call to arrival at the hospital was similar for non-chest pain and chest pain patients who received emergency ambulances (51.5 IQR[41.1;63.5] versus 46.4 IQR[37.2;56.6] minutes), but it varied for non-chest pain versus chest pain MI patients who did not receive emergency ambulances (61.6 IQR[44.2;92.7] versus 49.7 IQR[29.9;68.8] minutes). See Additional file 1: Table S5 for additional details. **B** Only MI patients who received an emergency ambulance and did not have contraindications of ASA were included in this figure; this was 4150 out of the 4214 MI patients who received an emergency dispatch. *ASA* Acetylsalicylic acid

In Fig. 2 the number of MI patients who received emergency ambulance and prehospital ASA treatment is shown for MI patients with and without chest pain, separately. The proportion receiving an emergency ambulance and ASA treatment varied across symptom presentation. Among MI patients with chest pain, 90% (3689/4109) received an emergency ambulance. This proportion was 40% (525/1309) among patients without chest pain (Fig. 2A). The crude 30-day mortality among MI patients with chest pain was 3% (116/4109) and 11% (143/1309) among MI patients without chest pain. In total, 4150 MI patients received an emergency ambulance and did not have contraindication of ASA. Among these, 73% (2668/3632) with chest pain and 37% (192/518) without chest pain received ASA (Fig. 2B).

### Main analyses

**Fig. 3:**
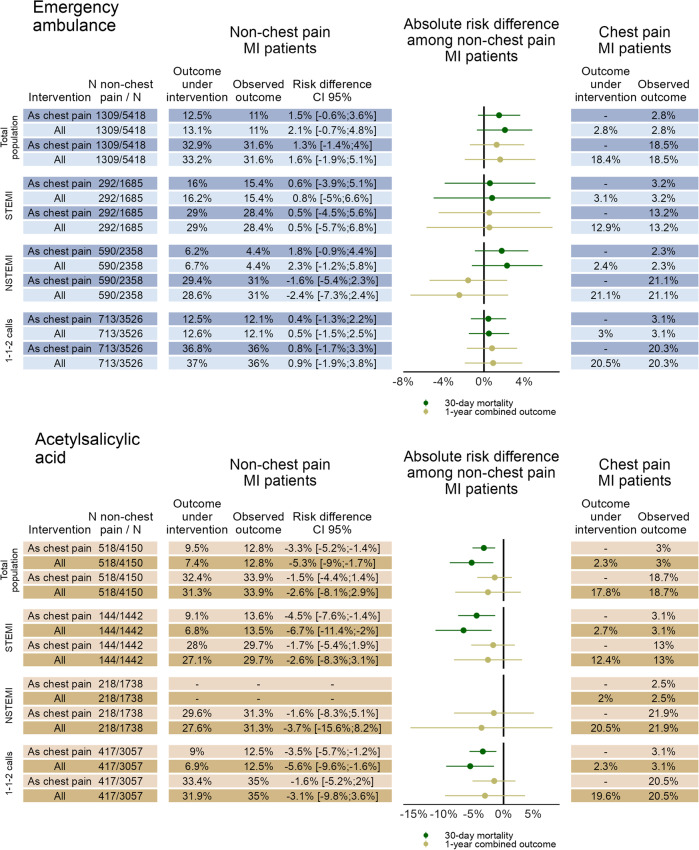
Expected change in outcome under hypothetical interventions on emergency ambulance dispatch and ASA administration. The figure shows the results of the hypothetical interventions on emergency ambulance dispatch and acetylsalicylic acid administration on 30-day mortality and 1-year combined-outcome among MI patients recorded without chest pain. The results are shown for the total populations and three subgroups (STEMI, NSTEMI, and 1-1-2 emergency calls). Observed outcomes and the expected outcomes had all received emergency ambulances/ASA are also shown for chest pain MI patients (right columns). The intervention *As chest pain* refers to the hypothetical intervention under which non-chest pain MI patients were as likely to receive emergency dispatch/ASA as observed for the MI patients with chest pain. Similarly, the *All* intervention refers to the hypothetical intervention where all non-chest pain MI patients received emergency ambulance/ASA. *ASA* Acetylsalicylic acid

In Fig. 3, the results of the hypothetical interventions on emergency ambulance and ASA on 30-day mortality and 1-year combined-outcome are shown for MI patients without chest pain and for the three subgroups (STEMI, NSTEMI, and 1-1-2 emergency calls).

In the main analysis of the hypothetical intervention on emergency ambulance, 5418 MI patients were included of which 1309 (24.2%) were recorded without chest pain. The observed 30-day mortality among MI patients without chest pain with no hypothetical intervention was 11% and 1-year combined outcome was 31.6%. In comparison, the observed 30-day mortality without intervention among MI patients recorded with chest pain was 2.8% and the 1-year combined outcome was 18.5%. We found a small non-significant increase for both 30-day mortality and 1-year combined outcome, with 30-day mortality increases ranging from 1.5% CI 95%[− 0.6%; 3.6%] (*as chest pain-intervention*) to 2.1% CI 95%[-0.7%; 4.8%] (*all-intervention*) and similarly for 1-year combined outcome ranging from 1.3% CI 95%[− 1.4%; 4%] *(as chest pain-intervention*) to 1.6% CI 95%[-1.9%;5.1%] (*all-intervention*).

For the analysis of the hypothetical intervention on prehospital ASA, 4150 MI patients who had received emergency ambulance and had no contraindications of ASA were included. Among these, 518 (12.5%) were recorded without chest pain. The observed 30-day mortality without intervention among non-chest pain MI patients was 12.8% and 1-year combined outcome was 33.9%. Similarly, among MI patients with chest pain, the observed 30-day mortality and 1-year combined outcome without intervention was 3% and 18.7%, respectively. Under the hypothetical intervention where non-chest pain MI patients had the same probability of receiving ASA as those with chest pain, 30-day mortality was reduced with 3.3% CI 95%[1.4%;5.2%]. The reduction increased to 5.3% CI 95%[1.7%;9%] for the intervention where all non-chest pain MI patients hypothetically received ASA. No significant changes were found for the hypothetical intervention on ASA for the 1-year combined outcome.

### Subgroup analyses

To investigate the impact of the type of MI on the results, we repeated the main analyses of the two hypothetical prehospital interventions; emergency ambulance and ASA in subpopulations of STEMI and NSTEMI patients. Additionally, subgroup analyses for calls to the 1-1-2 emergency number were performed to assess whether the effects of the prehospital interventions differed according to the choice of medical service.

For the subgroup analysis of the hypothetical intervention on emergency ambulance, we included 1685 STEMI patients, 2358 NSTEMI patients, and 3526 patients who had called the 1-1-2 emergency number (Fig. 3). For the subgroups, STEMI and emergency calls (1-1-2), we found no change in either 30-day mortality or 1-year combined outcome for the hypothetical interventions on emergency ambulance. For the NSTEMI population findings aligned with the total population, but for the 1-year combined outcome a small, although non-significant, decrease was found of 1.6% CI 95% [− 2.3%;5.4%] (*as chest pain-intervention*) and 2.4% CI 95% [− 2.4%;7.3%] (*all-intervention*). In the subgroup analysis of the hypothetical intervention on prehospital ASA, we included only emergency ambulance transported MI patients without contraindication of ASA. Here, the subgroups consisted of 1442 STEMI patients, 1738 NSTEMI patients, and 3057 patients who had called the 1-1-2 emergency number (Fig. 3). In the subgroups of both STEMI and 1-1-2 emergency calls results were similar to the total population. The largest reduction in 30-day mortality of 6.7% CI 95%[2%;11.4%] was found for non-chest pain STEMI patients if they hypothetically all had received prehospital ASA. Lack of data hindered analysis of the 30-day mortality among NSTEMI patients and uncertainty was large for the 1-year combined outcome.

## Discussion

Among the 24.2% (1309/5418) MI patients who were recorded without chest pain, the observed 30-day mortality and 1-year combined outcome to 11% and 31.6%, respectively. Large differences in the prehospital management of MI patients with and without chest pain exists, where 90% of chest pain versus 40% of non-chest pain patients received emergency ambulances, and 73% and 37% of chest pain and non-chest pain patients received prehospital ASA. We found no improvement in 30-day survival when hypothetically increasing chance of receiving emergency ambulance dispatch to all non-chest pain MI patients. Increasing prehospital administration of ASA to emergency ambulance transported non-chest pain MI patients was found to reduce 30-day mortality by 3.3% CI 95%[1.4%;5.2%] to 5.3% CI 95%[1.7%;9%] depending on the intervention. However, no significant reduction was found for the 1-year combined outcome when hypothetically increasing chance of receiving ASA in the prehospital setting. Despite the reductions found for the hypothetical interventions on ASA, 30-day mortality remained two to three times higher among non-chest pain MI patients compared to chest pain.

Contrary to our hypothesis, the hypothetical intervention on the probability of receiving an emergency ambulance did not change the risk of 30-day mortality and 1-year combined outcome. In fact, increasing emergency ambulance dispatch seemed to increase outcome likelihood slightly. The finding could be explained by the fact that MI patients with more severe symptoms are more likely to receive an emergency ambulance. These patients are equally more likely to have a more severe or progressed MI probably affecting the chance of survival. Unfortunately, we do not have data that can help us differentiate the severity of the patient's infarct at the time of the call.

We found a relatively large reduction in the risk of 30-day mortality among non-chest pain MI patients when hypothetically increasing prehospital ASA assignment. We cannot determine the exact cause of this reduction. It is possible that the reduction is at least partly caused by the effect of ASA, but we also expect that other factors such as early diagnostics influence the result. Providing ASA to a patient indicates that the ambulance rescue team has suspected MI and initiated a multi-faceted strategy including recording a prehospital electrocardiogram (ECG) and possibly consulting the cardiologist on duty at the invasive cardiac centre, which in addition increases the patient's chances of receiving timely in-hospital treatment. Non-chest pain MI patients are less likely to be referred directly to a cardiac centre and have previously been found to have a lower chance of receiving ASA as well as other medications within 24 h of hospitalization, indicating that they are likely to also experience in-hospital delays in treatment [4, 25]. Secondly, although prehospital administration of ASA is recommended to both NSTEMI and STEMI patients, the evidence for prehospital versus in-hospital administration is sparse, especially for the NSTEMI patients [8]. As just 27.8% (144/518) of emergency ambulance transported non-chest pain MI patients had STEMI, and thus, the vast majority of these patients had NSTEMI or other/unknown types of MI, the estimated reduction in 30-day mortality seems high. Additionally, distances to hospitals in Denmark are generally short with 36% of the Danish population having less than 10 km to the nearest hospital and just 3% having driving distances above 50 km [34]. Given the short distances and the lack of evidence of prehospitally administered ASA for especially NSTEMI patients, we would expect a relatively modest effect of assigning ASA prehospital versus in-hospital, and thus, residual confounding is expected to be affecting the results.

We found a small but not statistically significant reduction for 1-year combined outcome under the hypothetical interventions on ASA administration, and the expected 1-year combined outcome remained around 1.5 to 2 times higher among non-chest pain MI patients compared to chest pain MI patients. The differences in outcomes are expected to, at least partly, be explained by dissimilarities in age, comorbidities, and educational level. A larger comorbidity burden and lower educational level among non-chest pain MI patients was anticipated, as they were older. However, research have indicated that people’s knowledge of MI symptoms vary according to educational level [36], and we believe that both knowledge of MI symptoms and the ability to understand and communicate symptoms can affect how call takers perceived and recorded the symptoms.

The Danish guidelines are in accordance with the international guidelines and advocate prehospital administration of ASA to all eligible patients suspected of acute coronary syndrome [37]. In this study, only 73% and 37% of chest pain and non-chest pain MI patients transported with emergency ambulances received ASA indicating that especially non-chest pain, but also to some extent chest pain MI patients, receive suboptimal prehospital treatment. A previous study found that up to ~ 25% of chest pain patients had either self-administered ASA or received ASA by another health professional prehospital [38]. We do not have data on whether patients had received ASA before ambulance arrival, and this could at least partly explain why not all patients received ASA during ambulance transport as guidelines recommend. It is equally likely that there is some extent of under reporting of ASA administration by the ambulance personnel, but we believe over reporting is unlikely. The proportions of MI patients that received ASA were large in this study compared to previous findings, where 45–58% of acute coronary syndrome/MI patients received prehospital ASA [9, 14, 15]. Increased focus on prehospital care of acute coronary syndrome patients and direct transferring of prehospital ECGs to cardiologist might partly explain the higher prevalences.

### Limitations

In this study, the criterion recorded at the Copenhagen EMS was used as a proxy of the primary symptom. However, because patients can develop chest pain between the time of call and ambulance arrival, we do not know if the non-chest pain MI patients who were administered ASA received it because they developed chest pain or because of prehospital diagnosis with ECG.

The study population, covariates, and outcomes were defined using data recorded in the Danish national registers. We cannot rule out that coding errors and imprecise or missing registrations can affect the validity of the data [39, 40]. Further, it should be noted that the National Prescription Register only contains data on redeemed prescriptions [21], thus, we anticipate that we have underestimate the actual NSAID use as we do not include NSAIDS bought over the counter.

MI patients included in this study were diagnosed in-hospital. Thus, identification of these patients is not possible at the time of their call, and as a result, the hypothetical interventions of emergency ambulances and ASA treatment among MI patients examined in the present study cannot be identified in a real-world setting, hindering a direct causal interpretation of the results. Identification of MI patients based on symptoms alone is not sufficient, as non-chest pain MI patients present with a variety of symptoms. Broader use of ECG and point-of-care cardiac necrosis markers could possibly improve identification of MI patients during ambulance transport, and thus, be a tool for selecting patients for ASA administration, although we know very little about their effectiveness in patients presenting without chest pain.

A real-world intervention of emergency ambulance dispatch and prehospital ASA administration to suspected MI patients would also include dispatching ambulances and administering ASA to many patients not suffering an MI. Administration of ASA to patients not suffering from an MI could lead to severe adverse events including increased risk of bleeding. Likewise, increasing emergency ambulance dispatch would be expected to have economic and administrative consequences. Neither of these adverse effects were assessed in the present study. If this study was to be investigated in a real-world setting adverse effects of both increased emergency dispatch and ASA administration should be investigated thoroughly to be able to assess the cost versus benefits of the suggested prehospital interventions. Nevertheless, this study aimed to provide information on whether there is an indication of a possible benefit of improved prehospital management among the target population, namely MI patients presenting without chest pain.

### Implications

Compared to MI patients with chest pain, MI patients without chest pain had three times higher 30-day mortality and twofold increased 1-year combined outcome, regardless of any of the hypothetical interventions considered in this paper. Although some potential reductions in 30-day mortality were found when improving prehospital management, overall, MI patients recorded without chest pain still have a very poor prognosis. MI patients without chest pain had a higher prevalence of severe diagnosis during their admission including heart failure, COPD, and cardiogenic shock. The high prevalence of COPD aligns with the differences in comorbidity before the MI, but given that MI patients with and without chest pain had a similar prevalence of heart failure prior to the MI, the difference in heart failure diagnoses during the admission is alarming. In combination, these findings indicate that MI patients without chest pain are truly high-risk patients.

Non-chest pain MI patients are difficult to identify prehospital and real-world interventions would inevitably include many patients not suffering from an MI. Thus, one should carefully weigh the potential risk of adverse effects, including bleeding risk, when increasing prehospital ASA administration. Bleeding is a serious complication and bleeding risk assessment of patients in the prehospital setting is important but might be challenging, as accurate risk discrimination remains difficult [41].

Additionally, one must consider the possible large increase in economic as well as human resources needed to increase emergency ambulance dispatch, ASA administration, and presumably prehospital diagnostics, given the absence of long-term benefits and the uncertainty of the true cause of the reduction in 30-day mortality discussed in this paper.

## Conclusion

We found large differences in the prehospital management of MI patients with and without chest pain. Non-chest pain MI patients have low chance of receiving an emergency ambulance dispatch when calling for help and do often not receive ASA during the ambulance transport, indicating a need for improved prehospital management of non-chest pain MI patients. We found that hypothetically increasing ASA administration could lead to reduced 30-day mortality among non-chest pain MI patients. Still, the potential effect of increasing ambulance dispatch and ASA administration appears limited as non-chest pain MI patients currently are very difficult to identify in the prehospital setting and possible unintended side effects of ASA (e.g. bleeding risk) might outweigh the benefits. Early diagnosis among those receiving ASA is expected to contribute to the magnitude of our results for increased ASA use and confounding cannot be ruled out. A randomized trial investigating the beneficial as well as adverse effects of prehospital administration of ASA is warranted. Additionally, further research is needed to determine whether identification of non-chest pain MI patients could be improved by alternative medical telephone consultation techniques or broader criteria for using prehospital ECG and point-of-care cardiac necrosis markers.

## Supplementary Information

**Additional file 1: Table S1** ICD-10 codes for comorbidities registered prior to MI diagnosis and for diagnoses registered during MI admission. **Table S2** ATC-codes for claimed prescribed medication used to define presence of disease and use of medication. **Table S3** ICD-10 codes for MI diagnoses. **Table S4** Subset of diagnosis registered during admission with MI (for which the patient was included in this study). **Table S5** Minutes from call to hospital arrival for subgroups of chest pain and non-chest pain and emergency response.

## Data Availability

Due to restrictions related to Danish law and patient privacy, the data used in the present study can only be made available through a trusted third party, Statistics Denmark. Data can only be examined in collaboration with an authorized Danish investigator. Request for access can be send to Professor Christian Torp-Pedersen, ctp@heart.dk.
