# Supplementary material for: Hypothetical interventions on emergency ambulance and prehospital acetylsalicylic acid administration in myocardial infarction patients presenting without chest pain
Source: BMC Cardiovasc Disord. 2022 Dec 22;22:562. doi: 10.1186/s12872-022-03000-1 (PMC9783974; doi:10.1186/s12872-022-03000-1)
Supplement: Supplementary file 1 — Additional file 1: Table S1 ICD-10 codes for comorbidities registered prior to MI diagnosis and for diagnoses registered during MI admission. Table S2 ATC-codes for claimed prescribed medication used to define presence of disease and use of medication. Table S3 ICD-10 codes for MI diagnoses. Table S4 Subset of diagnosis registered during admission with MI (for which the patient was included in this study). Table S5 Minutes from call to hospital arrival for subgroups of chest pain and non-chest pain and emergency response. [file 12872_2022_3000_MOESM1_ESM.pdf]

Supplementary material

Table S1 ICD-10 codes for comorbidities registered prior to MI diagnosis and for diagnoses registered during MI admission.

Disease	ICD-10 codes
**Myocardial infarction**	I21
**Ischemic heart disease**	I20, I22-25
**Congestive heart failure**	I11.0, I13.0, I13.2, I42.0, I42.6, I42.7, I42.8, I42.9, I50.0, I50.1, I50.2, I50.3, I50.8, I50.9
**Moderate/severe renal disease**	I12, I13, N00-N05, N07, N11, N14, N17-N19, Q61
**Chronic obstructive lung disease**	J44
**Cancer (excluding malign melanoma)**	C00-C42 and C44-C97
**Arterial fibrillation**	I48
**Cardiac arrest**	I46
**Cardiogenic shock**	R570

Comorbidities were defined as registrations of a primary or secondary diagnosis in the Danish National Patient Registry up to 5 years prior to the call. For diagnosis registered during the MI admission for which the patient was included in the study, we included all types of diagnosis. ICD-10: International Classification of Diseases, 10th revision.

Table S2 ATC-codes for claimed prescribed medication used to define presence of disease and use of medication

Disease/medicine	ATC codes
**Type 2 diabetes**	A10
**Hypertension**	α adrenergic blockers: C02A, C02B, C02C<br>Diuretics: C02DA, C03A, C03B, C03D, C03E, C03X, C02DA, C07B, C07C, C07D, C08G, C09BA, C09DA, C09XA52, C02L<br>Vasodilators: C02DB, C02DD, C02DG,<br>Beta-blockers: C07A-D, C07F<br>Calcium channel blockers: C07FB, C08, C08G, C09BB, C09DB<br>Renin-angiotensin system inhibitors: C09AA, C09BA, C09BB, C09CA, C09DA, C09DB, C09XA02, C09XA52
**NSAID**	M01A
**Opioids**	N02AA01, N02AA03-5, N02AA55, N02AB02, N02AB03, N02AE01, N02AG02, N02AX02, N02AX06, N07BC02, R05DA04

Table S3 ICD-10 codes for MI diagnoses

MI diagnosis	ICD-10 codes
**STEMI**	I210B, I211B, I213
**NSTEMI**	I210A, I211A, I214
**Unknown/other**	I210, I211, I219, I219A

ICD-10: International Classification of Diseases, 10th revision.

Table S4 Subset of diagnosis registered during admission with MI (for which the patient was included in this study).

	Non-chest pain		Chest pain	
**Diagnosis registred during admission with MI**	N	Proportion	N	Proportion
**MI**	1309	100%	4109	100%
**Ischemic heart disease**	386	29.49%	1329	32.34%
**Heart failure**	282	21.54%	422	10.27%
**Atrial fibrillation**	138	10.54%	288	7.01%
**COPD**	126	9.63%	91	2.21%
**Moderate/severe renal disease**	68	5.19%	97	2.36%
**Cardiac arrest**	31	2.37%	46	1.12%
**Cardiogenic shock**	28	2.14%	25	0.61%

N is the number of admissions where the diagnosis was registered for non-chest pain and chest pain, respectively. The proportion indicates the share of all admissions for non-chest pain/chest pain where the diagnosis was registered. We included all types of diagnoses including primary, secondary, and referral diagnoses.

Table S5 Minutes from call to hospital arrival for subgroups of chest pain and non-chest pain and emergency response.

Population	Response	N	Time from call to hospital arrival in minutes				
			Mean	Median IQR [25%;75%]	10th percentile	90th percentile	Missing data
**Non-chest pain**	Emergency ambulance	527	57.6	51.5 [41.1;63.5]	34.1	78.5	4
	No emergency ambulance	782	124.0	61.6 [44.2;92.7]	27.2	199.5	4
**Chest pain**	Emergency ambulance	3731	49.3	46.4 [37.2;56.6]	30.6	69.9	14
	No emergency ambulance	378	92.7	49.7 [29.9;68.8]	16.4	99.4	≤ 3
